# Epigenetic Regulation of Key Enzymes CYP7a1 and HMGCR Affect Hepatic Cholesterol Metabolism in Different Breeds of Piglets

**DOI:** 10.3389/fvets.2020.00231

**Published:** 2020-05-15

**Authors:** Xian Li, Hanyang Xiao, Xiaoqian Jian, Xiangyin Zhang, Hui Zhang, Yang Mu, Hua Wang, Shulin Chen, Rihua Cong

**Affiliations:** ^1^College of Veterinary Medicine, Northwest A&F University, Shaanxi Yangling, China; ^2^Shaanxi Animal Health and Slaughter Management Station, Shaanxi Xi'an, China

**Keywords:** piglets, cholesterol metabolism, liver, epigenetic regulation, CYP7a1, HMGCR

## Abstract

Liver is the place where cholesterol is synthesized, transported, secreted, and transformed, thus liver takes an irreplaceable role in cholesterol homeostasis. Hepatic cholesterol metabolism differs between breeds, yet the molecular mechanism is unclear. In this study Large White (LW) and Erhualian (EHL) piglets (at birth and 25-day-old) were used, 6 each time point per breed. Erhualian piglets had significantly lower body and liver weight compared with Large White at birth and weaning, but the liver/ body weight ratio was higher at weaning, associated with increased serum and liver cholesterol and triglyceride content. The mRNA expression of Cholesterol-7alpha-hydroxylase *(CYP7a1)* and Recombinant Acetyl Coenzyme Acetyltransferase 2 (*ACAT2)* were down-regulated in Erhualian piglets at birth, while hepatic Sterol-regulatory element binding protein 2 (*SREBP2)* mRNA expression was up-regulated in Erhualian piglets at weaning, as well as SREBP2 protein content, compared with Large White piglets. At birth, the depressed CYP7a1 transcription in Erhualian piglets was associated with decreased Histone H3 (H3) and increased Histone H3 lysine 27 trimethylation (H3K27me3). While the results revealed significant promoter hypermethylation of 3-Hydroxy-3-methylglutaryl-CoA reductase *(HMGCR)* promoter in Erhualian piglets at weaning, together with increased Histone H3 lysine 9 monomethylation (H3K9me1) and Histone H3 lysine 4 trimethylation (H3K4me3). These results suggest that epigenetic modification may be an important mechanism in hepatic cholesterol metabolism among different species, which is vital for maintaining cholesterol homeostasis and decreasing risk of cardiovascular disease.

## Background

Cholesterol participates in the formation of cell membrane (1) and can synthesis bile acid and vitamin D as a precursor, which is of great significance to the life activities of the body (2, 3). Liver cholesterol content is determined by the net balance of cholesterol synthesis, transport and catabolism, i.e., cholesterol can be converted into bile acids (4). HMGCR is the key enzymes in cholesterol biosynthesis (5). HMGCR transcription is regulated by Sterol-regulatory element binding proteins (SREBPs), of which SREBP2 is the major subtype that regulates liver cholesterol (6, 7). Meanwhile, the content of cholesterol in the cell can affect the activity of HMGCR and SREBP2, thereby forming a feedback loop that forms the homeostasis of cholesterol (8). The transport of cholesterol is that into and out of the liver. Low-density lipoprotein (LDL) molecules are the main operator of cholesterol in the blood which are responsible for transporting liver cholesterol to other peripheral tissues, while High-density lipoprotein (HDL) transports cholesterol from extrahepatic tissues back to the liver. Low-density lipoprotein receptor (LDL-R) mediates the uptake of low-density lipoprotein cholesterol (LDL-C), which is very important in regulating serum concentrations of total cholesterol (Tch) and LDL-C. The key enzyme of cholesterol synthesis of bile acid in liver through the predominant pathway is CYP7a1 (9), however Cholesterol-27alpha-hydroxylase (CYP27a1) is the rate-limiting enzyme in the alternative pathway of bile acid synthesis.

The content of cholesterol in liver and serum is different vary from species to species and even within the same species. Studies have shown that the serum cholesterol content of different breeds of pigs was different (10, 11). Serum cholesterol concentration was lower in Full blood Chianina compared with crossbreds (Hereford × Angus crossbred), which was affected by sex class and dietary (12). There are significant differences in the sensitivity of different breeds of rabbits to hypercholesterolemia caused by a high cholesterol diet. Polymorphisms at genetic loci may be associated with significant heterogeneity in sensitivity to dietary cholesterol. These polymorphisms include absorption of dietary cholesterol, conversion of liver cholesterol to bile acids, feedback inhibition of endogenous cholesterol synthesis, or regulation of the LDL-R pathway (13–15). The expression level of *LDL-R* mRNA was different in muscle of pigs from different genetic groups, which was correlated with Intramuscular fat of longus dorsi (IMF) content in the Longissimus dorsi muscle of pigs (16). Indeed, while there are many reports about the difference of liver cholesterol content among varieties, molecular mechanisms of the difference were seldom studied.

HMGCR is essential for cholesterol synthesis. CYP7a1 is the rate-limiting enzymes in the classical pathway of bile acids synthesis in liver (17). Epigenetic regulation including DNA methylation and histone modification on fetal gene expression during cholesterol metabolism have been well-demonstrated (18). In angiocardiopathy and cancer researches, *CYP7a1* and *HMGCR* gene are described to be vulnerable to epigenetic regulation including DNA methylation and histone modification (18). However, whether interspecific differences affects gene expression via epigenetics has not been well-explored. Meaney provide a clue that DNA acylation has a key role in the overall coordination of cholesterol homeostasis (19). Different species modifies hepatic cholesterol metabolic gene transcription by DNA methylation and histones modification like H3K4me3, H3K27me3, and H3K9me1 (19–21). Activation of the *CYP7a1* gene is associated with an increase in H3 acetylation and H3K4me3 and a decrease in H3K9me1 and H3K27me3. Meanwhile, the activation of HMGCR transcription is accompanied by an increase in H3 acetylation and a decrease in histones H3, H3K9me1, and H3K27me3 (21–24). Based on these studies, we hypothesized that epigenetic events have a crucial function in modifying liver cholesterol metabolism in Large White and Erhualian piglets.

## Materials and Methods

### Animal and Sampling

The experimental animals include Large White and Erhualian pig, and the animal experiments were performed in the Conservation and Breeding Farm at Jiangsu Polytechnic College of Agriculture and Forestry, Jurong, Jiangsu Province, China. LW and EHL piglets (at birth and 25-day-old) were used in the study, 6 each time point per breed. All piglets were killed and sampled after weighing. Serum specimens were prepared and stored at −20°C. Liver specimens were collected within 20 min of death, quickly frozen in liquid nitrogen and stored at −80°C until further analysis. Animal experiments were conducted under the guidance of the Animal Ethics Committee of Northwest A&F University, China.

### Cholesterol in Serum and Liver

Total cholesterol in serum and liver was measured using a commercial cholesterol test kit (AO10027, Jinma Biotechnology Co., Ltd., Wenzhou, China). Respective assay kits (006340 and 006328, respectively, Beijing BHKT Clinical Reagent Co., Ltd., Beijing, China) were used to detect the concentrations of LDL-C and High-density lipoprotein cholesterol (HDL-C) in the serum. Liver cholesterol was extracted according to previously reported methods (Gibney and Nolan 2010). Briefly, 200 mg frozen liver sample was pipette in 1 ml of lysis buffer (18 mmol/L Tris, pH 7.5, 300 mmol/L mannitol, 50 mmol/L Ethylene Diamine Tetraacetic Acid(EDTA), 0.1 mmol/L PMSF) by a Polytron homogenizer (PT1200E, Brinkman Instruments, Littau, Switzerland). Two hundred microliter of homogenate were vigorously mixed with 800 μl chloroform/methanol (2:1, vol/vol) and centrifuged at 3,000 g for 5 min.

Cholesterol was extracted from the organic phase, air-dried and reconstituted in 30 μl of a mixture of tert-butanol and methanol (13:2, vol/vol). The cholesterol content was determined by cholesterol assay kit (Jinma Biotechnology Co., Ltd., Wenzhou, China).

### Total RNA Isolation and mRNA Quantification

Total RNA was isolated from liver samples using Trizol Reagent (Tiangen Biotech Co., Ltd., Beijing, China). Each sample was synthesized from total RNA using iScript cDNA Synthesis Toolkit (Promega, Madison, WI, USA) according to the manufacturer's instructions. Diluted 2 μl of cDNA (1:50) for real-time polymerase chain reaction (PCR). Primer sequences are shown in Table 1 and were synthesized by Invitrogen (Shanghai, China). Real-time PCR was performed in Mx3000P (Stratagene, USA). Simulated reverse transcription (RT) and no template controls were set to monitor the possible contamination of genomic and exogenous DNA both at RT and PCR. The specificity of amplification was determined by melting curve analysis and PCR product sequence.

**Table 1:** Primer sequences of the target genes.

**Target**	**Primer sequence (5^**′**^-3^**′**^)**	**Product (bp)**	**GenBank No**.
*SREBP1*	F: GCGACGGAGCCTCTGGTAGT	217	NM214157.1
	R: GCAAGACGGCGGATTTATTCA		
*SREBP2*	F: GCCTACCGCAAGGTGTTTC	305	DQ020476.1
	R: GTCATTTGCTGGCAGTCGTT		
*HMGCR*	F: CAGGCTGAAGTAAGGGAGA	174	DQ432054.1
	R: CACGAAGTAGGTGGCGAGA		
*LDL-R*	F: ACTGCTCATCCTCCTCTT	109	AF065990.1
	R: TTCCGTGGTCTTCTGGTA		
*LXR*	F: ATTTCCAGGAGTGCCGTCTT	102	AB254406.1
	R: CTTGCCGCTTCAGTTTCTT		
*FXR*	F: CGGAGAAGCATTACCA	137	XM 001928800.2
	R: AAGCATTCAGCCAACA		
*CYP7a1*	F: TATTCCTTCCGTTACCGAGTG	262	AK230868.1
	R: ACCTGACCAGTTCCGAGAT		
*CYP27a1*	F: TGTGGCTCGCATCGTTC	153	EF625352.1
	R: TCACCTGGCAGCTCCTT		
*GAPDH*	F: TACATGGTCTACATGTTCCAGTATG	285	DQ403065
	R: CAGGAGGCATTGCTGACAATCTTG		

### Tissue Protein Extraction and Western Blot Analysis

Extract total and nuclear proteins from 100 mg of frozen liver tissue, as previously described (30). Detection of protein concentration with Pierce BCA Protein Assay Kit (Thermo Scientific, USA). Western blot analysis for SREBP2 (ab30682, Abcam, UK, diluted 1:200) and HMGCR (sc-33827, Santa Cruz, USA, diluted 1:100) were follow the manufacturer's instructions. HMGCR and SREBP2 was normalized with Glyceraldehyde-3-phosphate dehydrogenase (GAPDH) (KC-5G4, Kangcheng, China, diluted 1:10,000).

### Chromatin Immunoprecipitation (ChIP)

ChIP experiment was performed according to the modified based on previous reports (37, 34). Briefly, 200 mg frozen liver sample was taken in liquid nitrogen, ground, and then resuspended in phosphate buffered saline (PBS) containing a mixture of protease inhibitors cocktail (Roche Diagnostics GmbH, Mannheim, Germany) and cross-linked in 1% formaldehyde for 10 min at room temperature. Then use 2.5 M glycine to stop the cross-linking reaction.

Washing the pellets with PBS and sodium dodecyl sulfate (SDS) lysis buffer (50 M Tris-HCl pH 8.1, 10 mM EDTA, 1% SDS containing protease inhibitors. The samples after cross-linked were sonicated for 10 min on ice with 10 s on/off intervals (Sonics Vibra, USA). To remove cell debris from the crude chromatin preparations the samples were then centrifuged at 12,000 rpm for 10 min at 4°C. According to the results of 1% agarose gel measurement, the average length of ultrasonic chromatin was about 500 bp. Prepare salmon sperm DNA/G protein agarose beads (60 μl, 50% slurry, Biyuntian, Biotechnology, China) in advance, dilute the Protein-DNA complex with ChIP dilution solution, and incubate 2 μg of the corresponding antibody at 4°C overnight [histone H3 antibody, ab1791, Abcam; anti-acetyl-histone H3, 06-599, Millipore; monomethyl-Histone H3K9 (Lys9) 17-680, Millipore; trimethyl-histone H3K27 (Lys27), 17-622, Millipore; trimethyl-histone H3K4 antibody, ab1012, Abcam]. Negative control group did not add antibodies. Add G protein agarose beads (120 μl slurry 50%) to capture chromatin immunoprecipitation complex. The pellets containing the immunoprecipitation complex were washed sequentially, and the antibody/protein/DNA complex were eluted from the protein G agarose beads. Finally, reversing cross-linking DNA fragments at 65°C for 5 h to purify the DNA that released from the immunoprecipitation complex. Immunoprecipitated DNA was used as a specific primer for the RT-PCR template to amplify the genomic sequence of the promoter regions of the *HMGCR* and *CYP7a1* genes.SREBP2 has no genomic DNA sequence, so this study did not use ChIP-qPCR to detect the SREBP2 promoter.

### Statistical Analysis

All data are expressed as mean ± SEM and were analyzed using independent-samples test with SPSS 13.0 for windows. The method of 2^−ΔΔ*Ct*^ was used to analyze the real-time PCR data. *P* < 0.05 is considered statistically significant.

## Result

### Body Weight and Cholesterol in Serum and Liver

**Figure 1:**
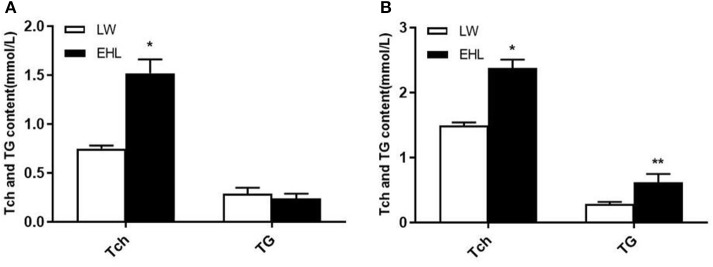
Total cholesterol and triglyceride content of the offspring piglets at birth **(A)** and weaning **(B)** (*n* = 6 piglets each time point per breed). *P < 0.05 and **P < 0.01. Data are presented as mean ± SEM.

As shown in Table 2, the piglets born from EHL sows had significantly lower body weight and liver weight at birth and weaning, while the ratio of liver weight to body weight (liver index) did not change at birth. The piglets born from EHL significantly increased liver index at weaning. This growth retardation is associated with significantly increased serum concentration and liver content of Tch (*P* < 0.05; Figure 1), HDL-C (*P* < 0.01), and LDL-C (*P* < 0.01) at birth and weaning.

**Table 2:** Body weight, liver weight, liver index, and the content of cholesterol in serum and liver of the offspring piglets at birth and weaning (*n* = 6 piglets each time point per breed).

**Parameters**	**LW**		**EHL**	
	**Birth (0 d)**	**Weaning (25 d)**	**Birth (0 d)**	**Weaning (25 d)**
Liver weight (g)	31.76 ± 1.72	178.74 ± 15.40	17.16 ± 1.21^**^	119.84 ± 6.72^**^
Body weight (kg)	1.31 ± 0.04	8.10 ± 0.34	0.75 ± 0.03^**^	4.08 ± 0.25^**^
Liver index (g/kg)	24.27 ± 0.91	21.92 ± 1.15	23.04 ± 2.01	29.51 ± 1.10^**^
TG (mmol/L)	0.29 ± 0.06	0.29 ± 0.03	0.24 ± 0.05	0.62 ± 0.128^**^
Tch (mmol/L)	0.75 ± 0.03	1.49 ± 0.05	1.52 ± 0.14^*^	2.38 ± 0.13^*^
HDL-C (mmol/L)	0.30 ± 0.02	0.47 ± 0.02	0.43 ± 0.03^**^	1.16 ± 0.08^**^
LDL-C (mmol/L)	0.23 ± 0.02	0.47 ± 0.01	0.52 ± 0.07^**^	0.76 ± 0.05^**^

* P < 0.05 and

** *P < 0.01. Data are presented as mean ± SEM*.

### Expression of Hepatic Genes Involved in Cholesterol Metabolism

**Figure 2:**
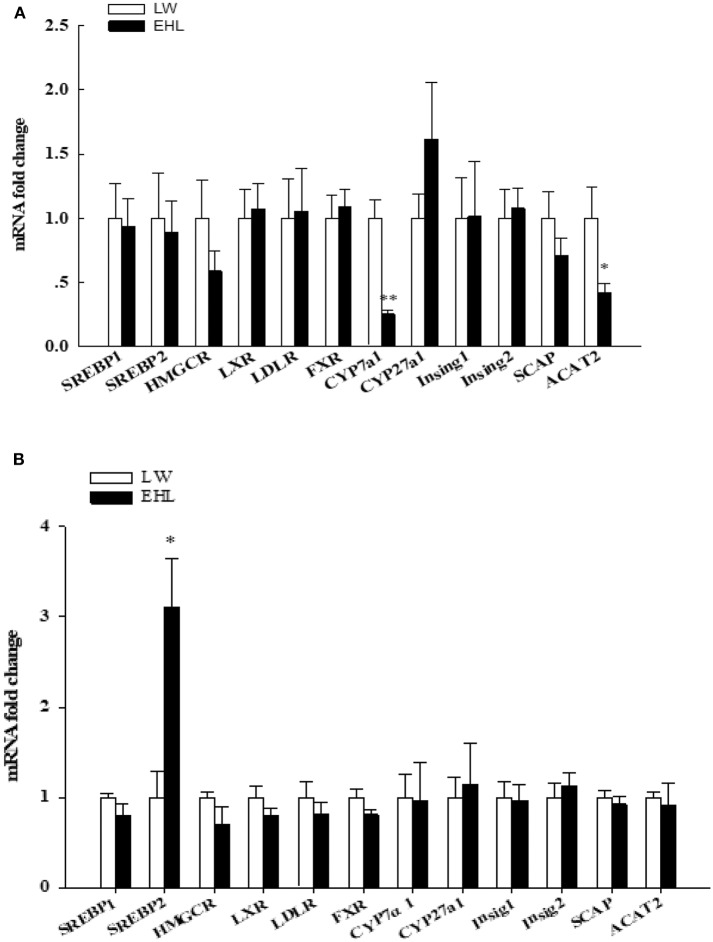
Hepatic expression of genes involved in cholesterol metabolism of the offspring piglets at birth **(A)** and weaning **(B)** (*n* = 6 piglets each time point per breed). *P < 0.05, **P < 0.01. Data are presented as mean ± SEM.

Using RT-PCR to quantitative detection mRNA abundance of 12 genes that involved in liver cholesterol metabolism. Among these genes, *ACAT2* (*P* < 0.05) and *CYP7a1* (*P* < 0.01) were found to be significantly down-regulated in the liver of EHL piglets at birth (Figure 2A). The levels of hepatic *SREBP2* mRNA in EHL piglets significantly higher than LW piglets at weaning (*P* < 0.05; Figure 2B).

### Hepatic SREBP2 and HMGCR Protein Content

**Figure 3:**
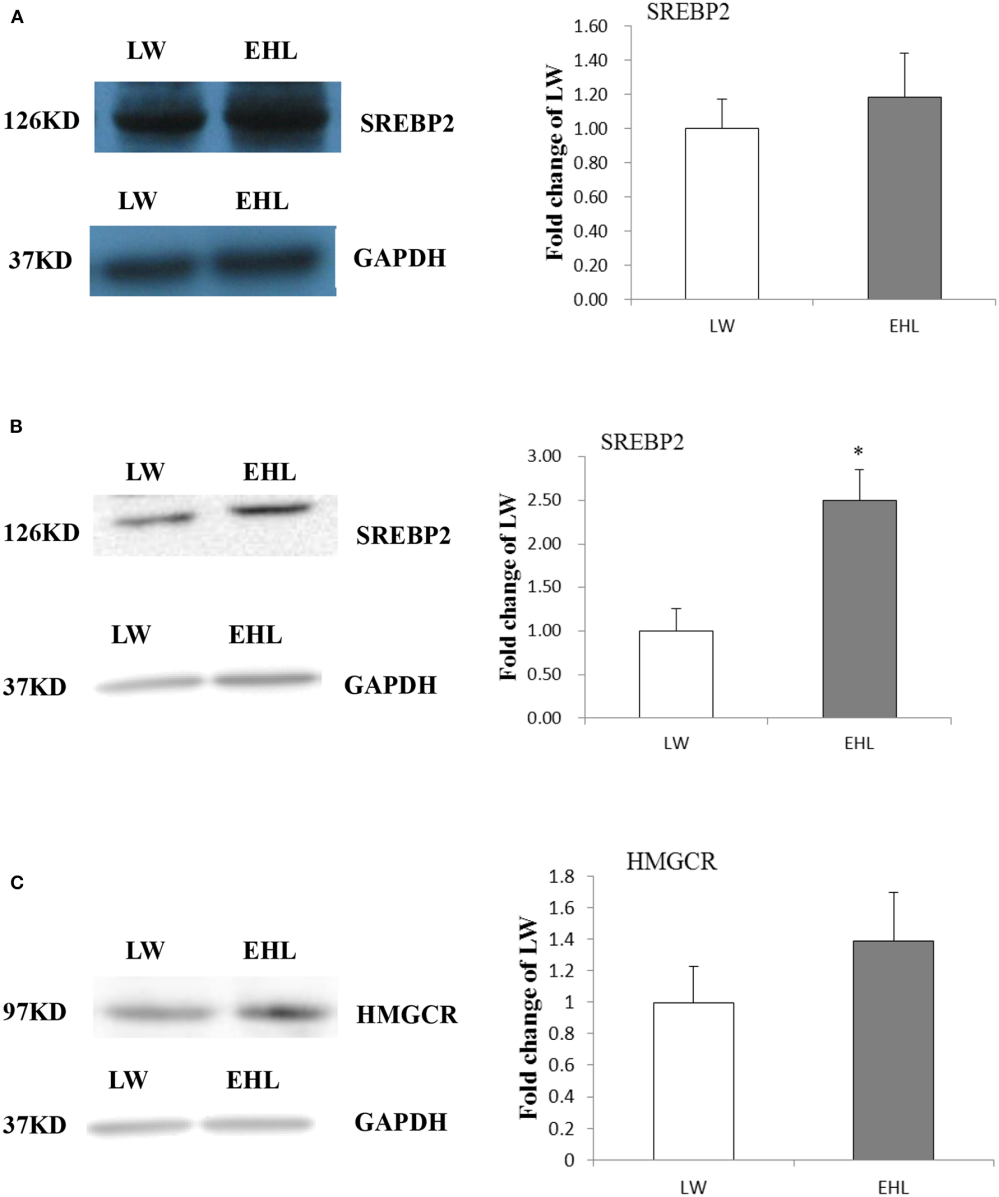
Hepatic SREBP2 and HMGCR protein content. **(A)** Expressed SREBP2 protein content at birth; **(B)** expressed SREBP2 protein content at weaning; **(C)** expressed HMGCR protein content at weaning (*n* = 6 piglets each time point per breed). *P < 0.05. Data are presented as mean ± SEM.

Western blot was used to detect the protein content of SREBP2 and HMGCR in the liver. The content of SREBP2 protein (Figure 3A, *P* > 0.05) at birth and HMGCR protein (Figure 3C, *P* > 0.05) at weaning was not affected by variety and age. However, the SREBP2 content in nuclear lysate was significantly increased (Figure 3B, *P* < 0.05) in the liver of EHL piglets at weaning, which was consistent with liver mRNA abundance.

### DNA Methylation

**Figure 4:**
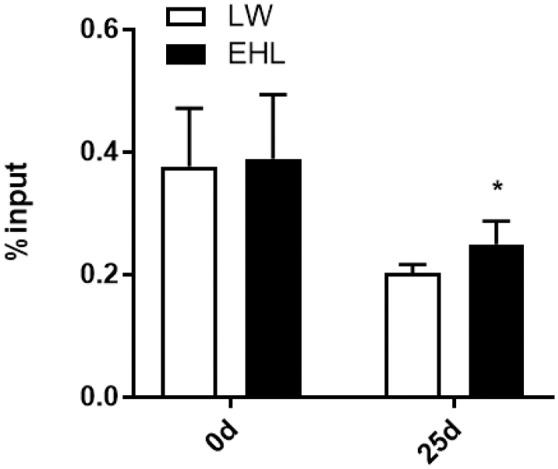
DNA methylation at *HMGCR* promoter in the liver of weaning piglets (*n* = 6 piglets each time point per breed). *P < 0.05. Data are presented as mean ± SEM.

MeDIP analysis showed that the HMGCR promoter was significantly hypermethylated (*P* < 0.05) in liver of EHL piglets at weaning (Figure 4). Because no CpG island was expected to exist in the CYP7a1 promoter, MeDIP analysis of the CYP7a1 promoter was excluded from this study.

### Histone Modifications

**Figure 5:**
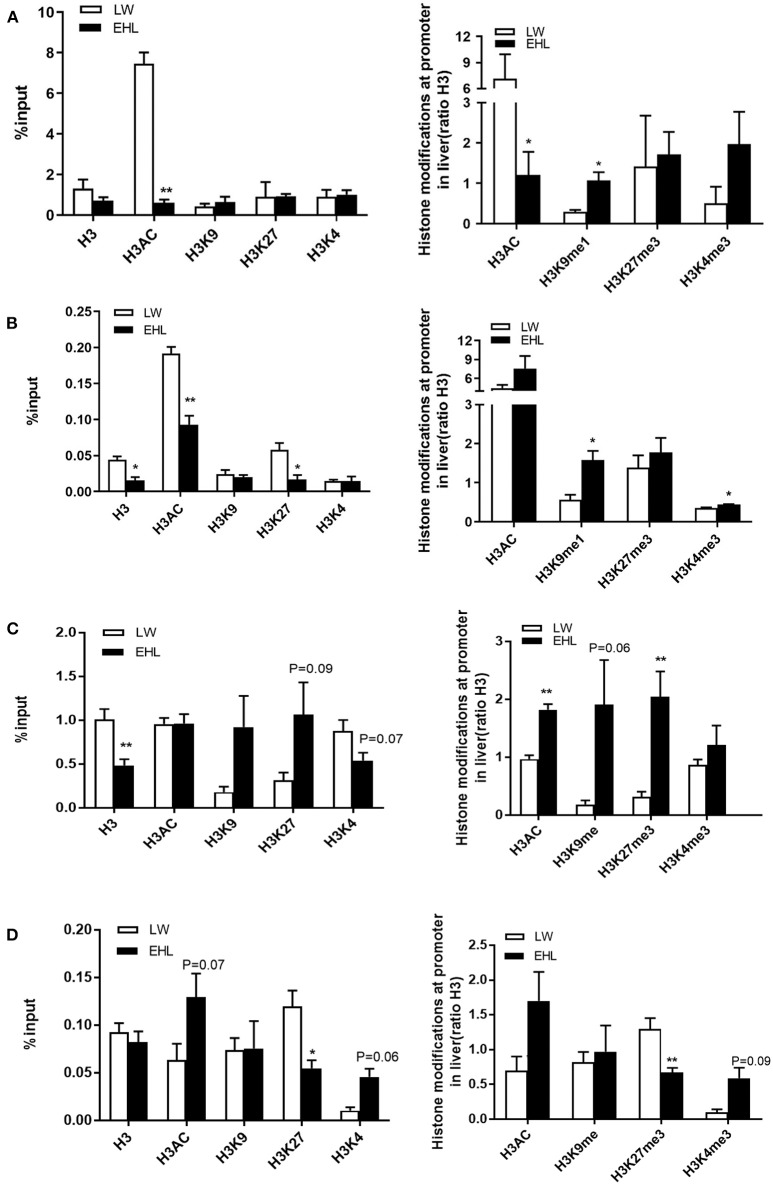
Histone modification at *HMGCR* and *CYP7a1* promoter in the liver of weaning piglets (*n* = 6 piglets each time point per breeds). **(A)** Expressed *HMGCR* as the percentage of the input and the ratio relative to H3 at birth; **(B)** expressed *HMGCR* as the percentage of the input and the ratio relative to H3 at weaning; **(C)** expressed *CYP7a1* as the percentage of the input and the ratio relative to H3 at birth; **(D)** expressed *CYP7a1* as the percentage of the input and the ratio relative to H3 at weaning. *P < 0.05 and **P < 0.01. Data are presented as mean ± SEM.

The enrichment of four histone modification marks, namely histone H3 acetylation (H3AC), histone H3 lysine 4 trimethylation (H3K4me3), histone H3 lysine 27 trimethylation (H3K27me3), and histone H3 lysine 9 monomethylation (H3K9me1), as well as histone H3 on the promoter of *HMGCR* and *CYP7a1*, analyzed by ChIP with specific antibodies. As shown in Figure 5A, when expressed as a percentage, the activation of hepatic *HMGCR* gene transcription in EHL piglets at birth was associated with a 685.1% decrease in H3AC (*P* < 0.01). When expressed as a ratio relative to H3, the transcription of *HMGCR* gene was related to the decrease of H3AC (*P* < 0.05) by 593.8%, and increase of H3K9me1 (*P* < 0.05) by 77.5%. Figure 5B showed significant decrease in histone H3 (*P* < 0.05; −2.9%), K27H3 (*P* < 0.05; −4.13%), and H3AC (*P* < 0.01; −9.83%) at weaning, expressed as percentage of the input, yet significant increase in H3K9me1 (*P* < 0.05; 102.1%) and H3K4me3 (*P* < 0.05; 8.57%) in EHL piglets at weaning, when expressed as the ratio relative to H3.

Figure 5C shows the enrichment and the modified forms of histone H3 in the *CYP7a1* promoter. The decrease in *CYP7a1* transcription in EHL piglets at birth was related to the downward trend (*P* = 0.07) in H3K4 (−33.5%) and uptrend (*P* = 0.09) in H3K27 (75.3%) and significantly decreased (*P* < 0.01) total H3 (−52.2%), expressed as percentage of input. A significant increase (*P* < 0.01) of total H3AC (85.6%) and H3K27me3 (172.8%) and a trend of increase (*P* = 0.06) of H3K9me1 (172.4%) were seen at the CYP7a1 promoter in EHL piglets at birth when expressed as the ratio to total H3. While a trend of increase in H3AC (*P* = 0.07; 6.55%) and H3K4 (*P* = 0.06; 3.53%) and significantly decreased H3K27me3 (*P* < 0.05; −6.48%) were seen at the *CYP7a1* promoter in EHL piglets at weaning, expressed as percentage of input. But when expressed as the ratio relative to H3 (Figure 5D), H3K27me3 (−62.6%) significantly (*P* < 0.01) decreased but H3K4me3 (*P* = 0.09; 49.1%) had the trend of increasement at weaning. There was no significant change in H3K4me3 of the promoter *HMGCR* or *CYP7a1* when expressed as a percentage.

## Discussion

EHL piglets had lower body and liver weight (*P* < 0.01) compared with LW piglets in both ages, while the liver/body weight ratio was higher at weaning, associated with the variety of pigs. Large White pig belongs to the breed with excellent growth rate, large body shape and high fecundity, but Erhualian pig is contrary. We have presented evidences that serum concentration and liver content of Tch and HDL-C were higher in EHL piglets, which was in agreement with early findings. Pond et al. found that the different content of serum cholesterol and HDL in variety was passed on from generation to generation when the serum of four-breed swine population was measured (11). We found that serum concentration of LDL-C was lower at birth but higher at weaning in EHL, but the reasons for these changes were not fully understood.

Cholesterol synthesis in pigs predominantly occurs in the liver. Among the ileum, cerebrum, kidney, heart, liver, semitendinosus muscle, longissimus muscle, and subcutaneous fat, the liver was the only tissue showing significant difference in cholesterol content among pigs with high or low serum cholesterol concentrations, indicating that liver is important to regulate cholesterol balance in pigs (25). There are four main pathways for cholesterol metabolism in liver: the first is that the *de novo* cholesterol biosynthesis mediated by HMGCR pathway; the second is that CYP7a1 and CYP27a1 catalyze the synthesis of bile acids; the third is that HDL mediate reverse cholesterol transport and LDL-R mediate endocytosis; the last is that cholesterol secrete into bloodstream via LDL-C. It has been reported that *ACAT2* participate in cholesterol absorption, esterify dissociative cholesterol in cell, and reduce the toxic effect of high free cholesterol on cell (26–28).

Similarly, we demonstrated that the expression of *CYP7a1* and *ACAT2* were decreased in EHL at birth. Serao at al. found that different pig breeds and lines present different content of intramuscular fat (16). Because no significant change in mRNA expression in liver tissue of Isigs, *Sterol-regulatory element binding protein cleavage-activating protein (SCAP), Sterol-regulatory element binding protein 1* (*SREBP1), SREBP2, HMGCR*, or liver content of SREBP1 protein and HMGCR protein, we demonstrated that the liver cholesterol biosynthesis was not increased in both pig breeds at birth. While the cholesterol esterification via *ACAT2* and transformation via *CYP7a1* were lower in EHL at birth, which is further clarified the liver cholesterol transformation to bile acids, cholesterol absorption, and dissociative cholesterol esterification were reduced. At 25-day-old, the increase of liver cholesterol content in EHL piglets is related to the significant increase of liver SREBP2 mRNA expression and the increase of SREBP2 protein in liver nuclear lysate, but no difference in CYP7a1. Thus, the liver cholesterol metabolism was balanced by reversed cholesterol transport in EHL, but the liver cholesterol content was higher in EHL compared with LW. This might be explained by higher cholesterol biosynthesis and lower cholesterol transformation in liver.

Based on our and other precious studies, liver modulates cholesterol transport, biosynthesis and transformation in pigs predominantly via epigenetic regulation (20, 21). We chose *CYP7a1* and *HMGCR* for epigenetic because the promoter sequences of these two genes can be used in pigs and both of them are key enzymes in the cholesterol metabolism (20, 29, 30). CpG island cytosine methylation located in promoter genes is associated with gene suppression (31, 32), while histone acetylation is related to the activation of transcription (20, 29, 30). According to the type of histone, the position of amino acid residues and the number of methyl groups (mono-, di- and trimethylation), histone methylation can inhibit or activate gene transcription. H3K4me3 is generally considered an activation marker, while H3K9me1 and H3K27me3 have repressive effects on transcriptional (22–24). These views are confirmed in a representative study of transcriptional suppression of *CYP7a1* in EHL is associated with increase in activation markers, H3K9me1 and H3K27me3 at birth, but the increased H3 acetylation was due to decreased histone H3, which content varies with species (33–37). Since increased histone H3K9me1 and H3K4me3, together with up-regulated *SREBP2* mRNA expression, *HMGCR* gene should be activated, but no significant alteration was observed for the mRNA expression and protein content of HMGCR in EHL and LW at weaning. While the liver content of cholesterol was higher in EHL, we speculate that *HMGCR* differ in its susceptibility to cholesterol (38–42).

These results showed that different species or breeds have different cholesterol levels and metabolic. Besides, diet and environment have influence on cholesterol metabolism. Serao et al. studied the relationship between lipid content and gene expression in the muscle of three breeds of pigs, and found that the expression of LDL-R mRNA was different in different breeds of pigs. It suggests that endocytosis of cholesterol in muscle tissue was different (16). The study showed that yolk cholesterol content was not only related to the breed of laying hens, but also to the age of the hen (43). Literature indicates that fatty acid composition varies between breeds. Due to the higher oleic acid concentration, the proportion of monounsaturated fatty acids (MUFA) in marbled Wagyu and Hanwoo beef was higher. They can lower LDL-C while increasing HDL-C, and may reduce risk for cardiovascular diseases (44). Clinical studies have shown that, low concentrations of SFA C12: 0, C14: 0 are benefit and important for longissimus lumborum muscle of Boer crossbreed goats compared with Santa Inês breed sheep. They are promoting the accumulation of low-density lipoprotein, which increases the risk factors of cardiovascular disease in humans (45). A large number of experiments indicated that the cholesterol content had variety specificity. Environmental affects cholesterol metabolism, and this effect is different in specie and tissue. Recently, methylation is very important in the development of human cardiovascular diseases. With the rapid development of high-throughput methylation technology, it has made a great breakthrough in epigenomic research, other biological and clinical fields. Studies have shown that epigenetic regulation involved in cholesterol synthesis, absorption, elimination, and storage so related to total cholesterol levels. Even be involved in the regulation of lipid concentration variability, and leading to cardiovascular disease. As methylation level increasing, the risk of Coronary heart disease (CHD), and higher TG and LDL-C levels and lower HDL-C level result in the change of DNA methylation.

In conclusion, we showed that the liver content of cholesterol was higher, and the ability of cholesterol biosynthesis was stronger in EHL compared with LW, and the molecular mechanism for this difference is enzymatic regulatory and age-dependent. These results provide a new idea for the study of lipid metabolism and meat quality difference mechanism between Erhualian pigs and large white pigs, and provide a reference for the study of the cardiovascular diseases.

## Data Availability Statement

All datasets generated for this study are included in the article/supplementary material.

## Ethics Statement

This animal study was reviewed and approved by NWAFU.

## Author Contributions

XL and RC contributed to the conception of the study. HX and XZ contributed significantly to analysis and manuscript preparation. HX, XJ, and HZ performed the data analyses and wrote the manuscript. HW, YM, and SC helped perform the analysis with constructive discussions.

## Conflict of Interest

The authors declare that the research was conducted in the absence of any commercial or financial relationships that could be construed as a potential conflict of interest.
